# Frailty and Overall Survival of Older Patients Undergoing Radiotherapy for Head and Neck Cancer: A Prospective Analysis

**DOI:** 10.3390/cancers16233939

**Published:** 2024-11-25

**Authors:** Chiara Giannotti, Silvia Ottaviani, Mariya Muzyka, Luca Tagliafico, Almalina Bacigalupo, Liliana Belgioia, Celjeta Tominaj, Stefania Vecchio, Fiammetta Monacelli, Alessio Nencioni

**Affiliations:** 1 IRCCS Ospedale Policlinico San Martino, 16132 Genoa, Italy; 2 Geriatrics Clinic, Department of Internal Medicine and Medical Specialties (DIMI), University of Genoa, 16132 Genoa, Italy; 3 Radiation Oncology Department, IRCCS Ospedale Policlinco San Martino, 16132 Genoa, Italy; 4 Department of Health Science (DISSAL), University of Genoa, 16132 Genoa, Italy; 5 Medical Oncology Department, IRCCS Ospedale Policlinico San Martino, 16132 Genoa, Italy

**Keywords:** head and neck squamous cell carcinoma, older adults, radiotherapy, frailty, toxicity

## Abstract

Background/Objectives: Over 60% of diagnoses of head and neck squamous cell carcinoma (HNSCC) occur in patients aged over 65. The benefits of radiotherapy (RT) combined with chemotherapy remain controversial in geriatric patients. Frailty stratification is underutilized in clinical practice despite evidence suggesting its predictive power. We aimed to assess the impact of pre-treatment frailty stratification on overall survival (OS) and acute radiation-related toxicity in a cohort of older patients with HNSCC. Methods: A prospective cohort of 117 patients aged ≥65 years with newly diagnosed HNSCC was enrolled between 2017 and 2022. Patients received RT with or without chemotherapy. Frailty was assessed using the 40-item Frailty Index (FI) as part of a comprehensive geriatric assessment. The primary outcomes were OS and acute RT toxicity, defined as a grade ≥ 3 based on CTCAE 5.0. Cox proportional hazard models and logistic regression models, adjusted for age, tumor stage, and chemotherapy, were used. Results: After a median follow-up of 819 days, FI (HR 1.478, 95% CI 1.182–1.848, *p* < 0.001) was an independent predictor of OS. Grade ≥ 3 toxicities were observed in 38% of patients, but frailty was not significantly associated with toxicity. A sub-analysis of oropharyngeal carcinoma patients demonstrated improved OS in HPV-positive patients (HR 0.231, 95% CI 0.078–0.686, *p* = 0.008). Conclusions: the standardized introduction of frailty assessment in the evaluation of older HNSCC patients holds great promise for predicting long-term survival. This approach could be crucial in tailoring treatment protocols that account for the biological heterogeneity of older adults.

## Simple Summary

Head and neck squamous cell carcinoma (HNSCC) is a common cancer in older adults, but treatment decisions for these patients can be challenging due to age-related factors, such as frailty. This study aimed to evaluate how pre-treatment frailty assessment can help predict survival outcomes and treatment-related side effects in older patients with HNSCC who undergo radiotherapy, with or without chemotherapy. We found that frailty plays a significant role in determining overall survival, but it was not directly related to acute radiation toxicity. Our findings suggest that incorporating frailty assessment into treatment planning could help personalize therapy for older patients, improving decision-making and potentially enhancing long-term outcomes.

## 1. Introduction

Recent data suggest that more than 60% of all new neck squamous cell carcinoma (HNSCC) diagnoses occur in patients aged over 65, and nearly 24% of newly diagnosed patients are older than 70 years [1,2]. 

Radiotherapy (RT) plays a key relevant role in the curative treatment of HNSCC, either in combination with surgery or systemic treatment, or as a single therapeutic approach [3,4]. Its combination with anticancer agents has led to significant improvements in locoregional tumor control and overall survival [5,6], but data about the benefit of chemoradiotherapy in old-age patients are conflicting. 

Some studies have indicated a diminishing survival benefit from concomitant chemotherapy with advancing age. However, only 7.5% of patients in this meta-analysis were over 70 years old, limiting the generalizability of these results to the wider geriatric population [7]. On the other hand, several data emphasize that age alone may not be a contraindication to effective treatment because fit older patients have been shown to tolerate radical cancer treatment as well as younger patients [4,8,9,10]. Old-age patients undergoing combined radical treatment with RT and chemotherapy (CT) resulted in better loco-regional control rates than a less aggressive treatment with RT alone, without evidence of impaired treatment tolerance [11]. 

Managing HNSCC in older adults is particularly challenging, as inadequate assessment of this unique patient population can result in either undertreatment or excessively aggressive therapy, both of which may significantly hinder their recovery. 

Assessing the frailty of older adults with HNSCC for curative treatment is interesting due to the prevalence of comorbidity, nutrition deficits, inadequate social supports and/or impaired cognitive abilities [3,12,13]. These clinical vulnerabilities are poorly covered by routine oncological assessments, and Belgioia et al. showed that although newly diagnosed older patients with HNSCC were reviewed in a multidisciplinary setting to optimize treatment, frailty stratification was conducted in only 10% of radiotherapy–oncology departments [11]. 

Recent evidence demonstrated that frailty is closely related to treatment toxicity in HNSCC patients [14] and can predict quality-of-life outcomes among older adults with HNSCC receiving definitive treatment [15]. Furthermore, appropriate frailty stratification can inform medical decision-making processes [16] and help to tailor the intensity of therapy according to each patient’s expected treatment tolerance [17,18].

However, data reviewing appropriate patient selection, the optimal time of intervention, and which treatment modality to use are limited and heterogeneous in HNSCC older patients [12,13], and further research is required to fully understand the importance of geriatric assessment and frailty stratification in this particular population. 

Given this scenario, the present study sought to evaluate the impact of pre-treatment frailty stratification on overall survival and acute radiation-related toxicity in a cohort of older patients newly diagnosed with HNSCC receiving RT with or without chemotherapy. The null hypothesis of this study is that pre-treatment frailty stratification has no significant impact on overall survival (OS) or acute radiation-related toxicity in older patients with head and neck squamous cell carcinoma (HNSCC).

## 2. Materials and Methods

### 2.1. Study Design and Patient Selection

This prospective study was performed between the radiation oncology unit and oncogeriatric outpatient clinic at IRCCS Ospedale Policlinico San Martino, Genoa, Italy.

This study was approved by the IRB (CERA N 24-54) University of Genoa, Italy.

From July 2017 to November 2022, 119 older cancer patients, all candidates for radiation therapy for head and neck cancer, were consecutively enrolled.

The inclusion criteria were as follows:

- Patients aged 65 years or older;
- Histologically confirmed diagnosis of head and neck cancer;
- No prior active cancer treatments;
- Eligible for radiotherapy.

The exclusion criteria were as follows:

- Age under 65 years;
- Previous antineoplastic treatment or head and neck irradiation;
- Metastatic disease;
- Inability to provide valid informed consent.

Cancer subsites included the oropharynx, oral cavity, hypopharynx, nasal cavity and paranasal sinus, larynx, and salivary glands. In the oropharynx group, HPV status was confirmed by immunohistochemical evaluation performed with anti-p16-NK4a monoclonal antibody (MTM Laboratories, Heidelberg, Germany).

We collected data on the patients’ demographics, including sex, smoking, alcohol use, and age at the time of enrollment. 

The cancer stage was assessed using the 8th edition of TNM classification. All patients were discussed in multidisciplinary boards to determine the correct therapeutic approach.

Following radiation treatment, acute toxicity data were collected at the end of the treatment and categorized according to the CTCAE 5.0 scale [19]. For the purpose of subsequent statistical analysis, a toxicity level of at least grade 3 during the reporting period was considered significant. 

The primary outcome of survival analysis was OS. Overall survival data were collected through the regional health portal (ASL3, Genova, Italy, electronic health data) and updated until 15 June 2023.

### 2.2. Geriatric Assessment

Enrolled patients referred to the oncogeriatric clinic of the IRCCS Polyclinic San Martino Hospital, Genoa, Italy between July 2017 and November 2022. Each patient received a comprehensive geriatric assessment (CGA), which included the Barthel Index [20] and Instrumental Activities of Daily Living (IADL) [21] to assess functional status; the Mini-Mental State Examination (MMSE) [22] and Clock Drawing Test [23] to assess cognitive status; the Mini Nutritional Assessment (MNA) [24] to assess nutritional status; a 15-item Geriatric Depression Scale (GDS) [25] to assess mood; the number of medications to assess polypharmacy; the Cumulative Illness Rating Scale (CIRS) [26] to assess multimorbidity; hand grip (HG, using a GIMA 28791 Smedley dynamometer) to assess sarcopenia; Timed-Up and Go (TUG) [27] to assess physical performance; EuroQoL to measure their perceived quality of life [28]. 

The patients’ frailty status was assessed through the 40-item Frailty Index (FI) [29,30], which uses a deficit accumulation approach and includes self-reported items on medical history, such as comorbidities (arterial hypertension, myocardial ischemia, heart failure, stroke, cancer, diabetes, osteoarthritis, and pulmonary disease), mood disorder, impairment in ADLs and IADLs, cognitive dysfunction, measurement of physical capacity, and nutrition (weight loss and body mass index). These items were scored either a 0 (no deficit) or a 1 (the presence of a deficit). The FI score was calculated for each participant by summing the number of deficit parameters and dividing it by the total number of parameters. Based on the FI, three groups were devised: fit (≤0.08), pre-frail (0.9–0.24), and frail (≥0.25).

Supplementary Material S1 displays the complete preoperative CGA and frailty assessment, detailing the domains, number of items, range, and cut-off for each scale. Supplementary Material S2 contains an overview of the frailty assessment according to the deficit accumulation model and details of the used version of the FI.

### 2.3. Radiotherapy and Systemic Therapy

Treatment consisted of curative RT with or without chemotherapy or anti-EGFR (epidermal growth factor receptor) therapy, or adjuvant RT with or without systemic therapy after primary surgery. 

A planning CT scan was conducted with the patient positioned for treatment, utilizing an immobilization system composed of a 5-point thermoplastic mask covering the head and shoulders, with a 2.5 mm slice thickness. The gross tumor volume (GTV) was defined based on clinical evaluation, endoscopic examination, CT, MRI, or PET scan results. 

Intensity-modulated radiation therapy (IMRT) was applied following a standard fractionation regimen of 2 Gy per day, with a total dose of 70 Gy for definitive treatment and 60–66 Gy for adjuvant therapy. Radiation delivery was carried out using a high-tech linear accelerator (Clinac 2100 or Trilogy, Varian, Palo Alto, CA, USA) or Helical Tomotherapy (Accuray, Sunnyvale, CA, USA).

Chemotherapy with cisplatin (CDDP) was administered at a dose of 100 mg/m^2^ every 3 weeks or 40 mg/m^2^ weekly, in combination with radiation therapy, or cetuximab was used with radiation at the discretion of an oncologist specializing in HNSCC.

### 2.4. Statistical Analysis

The descriptive analysis for quantitative variables is expressed as the median and interquartile range (IQR). First, we explored, by means of logistic regression, the relationship between the geriatric assessment variables and the risk of developing at least grade 3 toxicity. All measures that were found to be significant in the univariate analysis (*p*-value < 0.10) were included in the multivariable model after adjusting for age, stage of cancer (according to the 8th edition of the TNM classification), and whether concomitant chemotherapy was given. 

The primary outcome was overall survival (OS), estimated by the Kaplan–Meier method and compared using a log-rank test; OS was defined as the time from the end of RT to death for dead subjects or to the censoring date of survivors. 

Secondly, Cox proportional hazards models were fitted to assess the effects of the geriatric variables on all-cause mortality. The models were stratified and adjusted for age, stage of cancer, and concomitant chemotherapy. The results are presented as the hazard ratios (HR) and 95% CI.

An additional multivariate analysis was also performed to exclude clinical variables with high collinearity.

Finally, a sub-analysis was performed on patients with carcinoma of the oropharynx to further investigate the association between HPV and survival.

All reported analyses were run by RStudio (Version 2022.07), with a two-sided α level of less than 0.05 considered statistically significant.

## 3. Results

### 3.1. Patient Demographic and Clinical Characteristics 

A total of 119 patients were enrolled, but 2 patients were excluded as they did not undergo geriatric evaluation. The baseline clinical characteristics of the 117 eligible patients are shown in Table 1.

The median age was 76 (range of 65–91 at first visit) years, and 74.4% of patients were male. The most prevalent cancer subsite was oropharyngeal (n = 60), followed by laryngeal (n = 22). The majority of patients had stage IV (88%) or stage III (16%) cancer. Approximately 37% of the patients underwent concomitant chemotherapy.

GA impairment was highly present and, overall, half of the patients had at least three impaired domains. 

The frailty stratification based on the FI showed that the majority (58%) of patients were pre-frail.

### 3.2. Outcomes

#### 3.2.1. Overall Survival Analysis

The mean number of days of follow up was 818.9 days (range of 66–2164). Cox proportional hazards models were fitted to assess the effects of the geriatric variables on overall survival. All models were stratified and adjusted for age, stage of cancer, and concomitant chemotherapy. 

The clinical variables significant in the univariate analysis (*p*-value threshold of 0.10) entered the first multivariate model that showed functional status (lost IADL and Barthel Index, with an HR of 1.451, 95% CI 1.087–1.936, and *p*-value of 0.012, and an HR of 0.919, 95% CI 0.850–0.995, and *p*-value of 0.036, respectively) as the major determinant of OS. A second multivariate model adjusted for collinearity was made maintaining only the FI, since the latter included the remaining geriatric variables. This model showed that the FI was the main determinant of OS (HR of 1.478, 95% CI 1.182–1.848, and *p*-value < 0.001) (Table 2).

#### 3.2.2. RT-Related Toxicity

**Figure 1:**
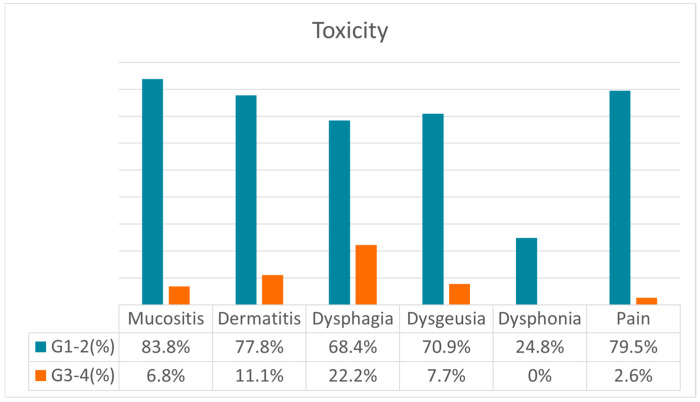
Different types of toxicities experienced after radiotherapy. Abbreviation list: G: grade of toxicity.

The majority (97%) of patients had multiple toxicities, although most of these were of grade 1 or 2. More significant toxicities (grades 3–4) occurred in 38% of patients. Few patients (9%) experienced multiple grade 3 toxicities. No grade 4 or 5 toxicity occurred in any patient (Figure 1).

All patients completed the radiation treatment, and interruptions due to toxicity were recorded in only four patients (3- and 4-day breaks in one and three patients, respectively).

Multivariable analysis adjusted for age, stage and concomitant chemotherapy showed no significant correlations among the CGA variables, including the FI and radiation-induced G3-G5 toxicity (Supplementary Materials S3).

#### 3.2.3. Sub-Analysis: HPV Status and OS

**Figure 2:**
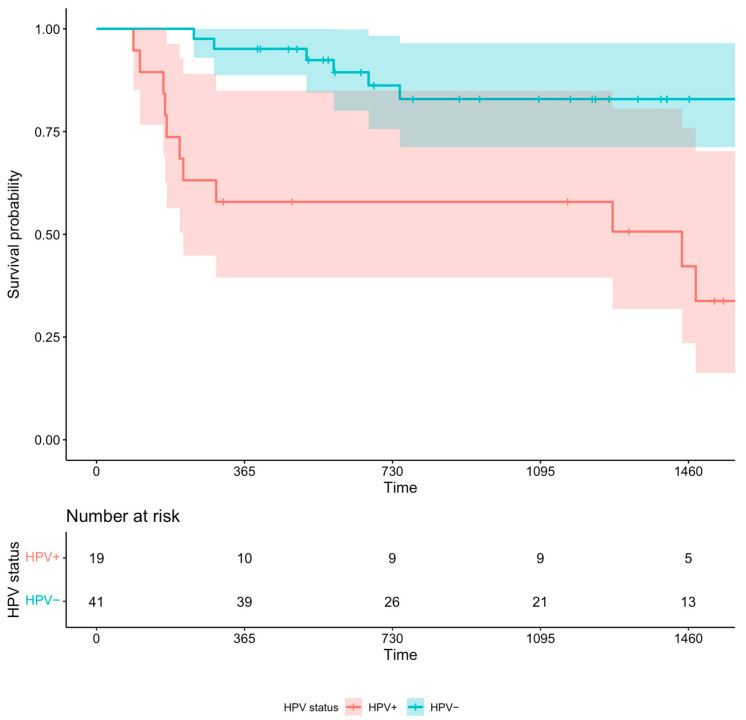
Kaplan–Meier survival analysis of 2 groups, HPV− and HPV+.

A total of 60 patients had oropharyngeal cancer; 41 of them were p16+. During the observation period, 19 patients died (8 HPV+). The Kaplan–Meier analysis of the two subgroups, shown in Figure 2, showed better survival in the HPV+ group, even though the confidence interval is very wide. The favorable prognostic impact of HPV positivity was confirmed by the multivariate Cox model, adjusted for age, concurrent chemotherapy, and tumor stage (HR of 0.2312, 95%CI 0.078–0.686, and *p*-value of 0.008). Furthermore, there was a trend for the HPV+ group to be less frail than the HPV- group, although it was not statistically significant.

## 4. Discussion

Geriatric HNSCC patients represent an extremely unique subset of patients, as demonstrated by the high prevalence of frailty syndrome [14,17]. Our study agrees with the prior literature reporting a high rate of frailty status, with the majority (58%) in the pre-frail category and 25% in the frail group. Pottel et al. found that the prevalence of frailty was 68.6% and 72%, based on the CGA, in patients referred for radiotherapy with and without chemotherapy for HNSCC, respectively [31]. In line with this, Bras et al. found frailty to be present in 40% of their study cohort [32]. 

These varied results may be attributed to differences in study populations, frailty assessment methods, and selection biases, making cross-study comparison difficult. Similar to our study, Siwakoti et al. utilized a deficit accumulation approach to develop a 44-item frailty index (CARE-FI), categorizing patients into robust, pre-frail, and frail groups. Approximately one-third of older adults were frail at baseline, which was independently linked to an almost threefold increase in mortality risk. These findings demonstrate that pre-treatment frailty has an adverse impact on long-term survival [33]. To our knowledge, our study is among the few prospective studies investigating the relationships between frailty assessment and overall survival in a cohort of older HNSCC patients receiving RT with or without chemotherapy. Moreover, our study confirms that the cancer stage, receiving chemotherapy in combination with RT, and the FI had a strong correlation with overall survival. 

Prior studies have shown that pre-treatment frailty is associated with adverse outcomes in the surgical setting, including increased post-operative surgical complications, acute medical complications, higher in-hospital death, longer length of stay, and increased costs after HNC surgery [34]. Kwon et al., in a study on both surgical and non-surgical HNC patients, showed an association between a revised frailty index that included respiratory and swallowing functions and 2-year mortality [35]. Furthermore, Pottel et al. [15] and de Vries reported [36] that in a cohort of HNC patients, frailty was associated with a worsening health-related quality of life.

The results of our study suggest that, in older HNSCC patients, incorporating frailty assessments using tools like the FI can provide critical information beyond traditional factors like age and cancer stage. A very recent study investigated the effects of a geriatric care pathway in HNC patients, including an abbreviated geriatric screening performed before the complete geriatric evaluation [37]. This two-step approach examined the proportion of patients referred to the geriatrician for CGA and the association between the screening tests and adverse outcomes. Likewise, our study offers new insights into the oncogeriatric pathway of care; in this case, HNSCC older patients referred to a CGA at the first presentation and frailty stratification seemed to provide important prognostic information in terms of overall survival. 

The referral of frail patients for CGA may help in the decision-making and tailored treatment; by pinpointing specific areas of frailty, clinicians can implement targeted interventions, such as optimizing nutritional status, managing comorbidities, or enhancing physical resilience through prehabilitation. This pre-treatment optimization could, in turn, improve patients’ overall tolerance to radiotherapy and potentially reduce treatment-related toxicities.

Indeed, an appropriate judgment of the biological age may also prevent the undertreatment of fit older patients and the overtreatment of the frail ones. Frail patients might benefit more from modified treatment protocols, such as altered fractionation schedules or reduced radiation volume. Notably, several ongoing clinical trials, including the NEVER trial (NCT: 0483255 [38]), are currently exploring this modified radiation approach, which could provide crucial insights into refining treatment protocols based on patients’ frailty status to propose a more tailored treatment.

The issue of treatment toxicity was investigated in our study, revealing that both FI and geriatric impairments are not associated with acute radiation-induced toxicity. Frailty is characterized by a decline in physiological reserves and homeostatic mechanisms in response to a stressful event. Curative radiation therapy is usually spread over 6 or 7 weeks, and this gradual increase in stress during the course of radiation treatment is probably better tolerated in frail patients. Our findings are in line with those of Pottel et al. [15], demonstrating that there was a trend, although statistically non-significant, indicating more severe toxicity and a higher need for hospitalization in vulnerable patients compared to fit patients. Similarly, in the studies by de Vries and Bras [32,36], frailty screenings using the Groningen Frailty Indicator (GFI) and Geriatric-8 (G8) were associated with adverse treatment outcomes in surgically treated patients, but not in patients undergoing radiation treatment. So, the length and intensity of stress could be an important factor. Surgery is an event that results in acute physical stress that is very intense at one time point, so a patient may run out of their physiological reserves more easily and would not be able to compensate [39]. 

Interestingly, considering the increasing incidence of human papilloma virus (HPV)-related oropharyngeal squamous cell carcinoma among patients aged 70 and older [40], we conducted a sub-analysis regarding HPV status in our cohort of older patients, showing that the HPV+ subgroup was associated with better clinical outcomes in term of overall survival, even in the analysis adjusted for age, concomitant chemotherapy, and cancer stage. Some evidence has underscored that HPV+ disease is most common in younger and healthier populations with low-grade morbidity, whereas the incidence, treatment tolerability, and outcomes in HPV-related oropharyngeal carcinoma in older individuals remain poorly defined [41]. Indeed, the sub-analysis revealed that older patients with HPV+ disease tended to be less frail than those with HPV-, as were younger populations. Despite the preliminary nature of these results and the very small size of the sample, our study confirms the prognostically favorable role of HPV+ status in oropharyngeal squamous cell carcinoma and takes a step toward future research that examines how this may guide multidisciplinary teams to modulate better treatment options. Larger, multi-center studies focusing on the interaction between HPV status and frailty could clarify whether an HPV-positive status consistently confers a survival benefit across varying frailty levels. This could further refine patient selection for combined therapies.

The strength of this study lies in the assessment of real-world geriatric HNSCC patients. Indeed, the study used a systematic assessment of frailty based on the deficit accumulation model. This multidimensional assessment has not been commonly used in previous studies, which have preferred less time-consuming frailty screening instruments. In addition, our present study was aimed at advancing our understanding of long-term clinical outcomes after RT treatment in old-age patients. 

The main limitations of our study are the use of a single-point frailty assessment and its monocentric design, which may introduce selection bias and limit the generalizability of our findings to broader HNSCC populations. Single-center studies can reflect specific institutional protocols, local patient demographics, and resource availability, which may not be representative of other settings. Future research would benefit from multicenter studies to capture a wider variety of clinical practices and patient populations, thus enhancing external validity. Additionally, incorporating repeated frailty assessments over the treatment course could provide a dynamic view of frailty status, capturing potential changes due to treatment or disease progression, and providing more understanding of how frailty influences outcomes across different healthcare settings. Moreover, the generalizability of our findings may be also limited by the inclusion of only patients aged ≥65 years, as this age threshold is commonly used in geriatric oncology studies. Future research could explore the variability across adjacent age groups to better understand treatment outcomes in a broader patient population.

## 5. Conclusions

In conclusion, although preliminary in nature, there is great potential for the standardized introduction of frailty assessment in the pretreatment evaluation of older HNSCC patients, representing an instrument able to provide reliable information on what to expect after cancer treatment in terms of long-term survival. Indeed, this approach could be of key relevance for tailoring protocols in older adults to guide choices of radiotherapy approaches based on a truly comprehensive vision of the older patient by virtue of their high biological heterogeneity. Eventually, targeting modifiable components of frailty could not only enhance treatment outcomes but also the quality of life of these patients, allowing them to better withstand the demands of curative cancer therapies.

## Figures and Tables

**Table 1:** The baseline clinical characteristics of the entire population. The results are presented as the median and interquartile range (IQR) and absolute frequency (n) and relative frequency (%) for the continuous and categorical variables, respectively, unless otherwise specified.

Variable	Value n = 117	IQR (0.25–0.75)
Sex (male)	87 (74.4%)	
Age at diagnosis, y (median)	76	72–81
Smoking (yes vs. no) Smokers, PY (median)	87 (74.4%) 38	4.5–70
Cancer type Oropharynx Oral cavity Hypopharynx Nasal cavity and paranasal sinus Larynx Salivary glands Unknown primary tumor	60 (51.3%) 13 (11.1%) 9 (7.7%) 4 (3.4%) 22 (18.8%) 1 (0.9%) 8 (6.8%)	
TNM staging T (1–2) T (3–4) N (0–1) N (2–3)	35 (29.9%) 82 (70.1%) 44 (37.6%) 73 (62.4%)	
Tumor stage 1 2 3 4	5 (4.3%) 5 (4.3%) 19 (16.2%) 88 (75.2%)	
Concomitant chemotherapy	43 (36.8%)	
Purpose of RT Curative Adjuvant	96 (82%) 21 (18%)	
Geriatric assessment (median)		
MMSE (n = 116)	27.6	26–29
CDT (n = 112)	2	1–4
MNA	23.5	21–25
IADL Lost IADL (≥1)	8 34 (29.1%)	5–8
Barthel Index Barthel Index ≤ 90	100 19 (16.2%)	95–100
CIRS Severity Comorbidity	1.9 4	1.7–2.1 3–5
GDS (n = 113) GDS > 5	3 17 (15%)	1–5
TUG (n = 106) TUG ≥ 15”	8.3 10 (9.4%)	7–10
HG (n = 107) M F	27 30 18.3	21.9–35.6 25.4–37.4 16.1–20.9
N of drugs	5	2.8–7
EuroQol (n = 106)	0.78	0.68–0.85
FI Fit (≤0.08) Pre-frail (0.09–0.24) Frail (≥0.25)	0.16 20 (17.1%) 68 (58.1%) 29 (24.8%)	0.10–0.25

Abbreviation list. TNM staging: Tumor, Node, and Metastasis staging; MMSE: Mini-Mental State Examination; CDT: Clock Drawing Test; MNA: Mini Nutritional Assessment; IADL: Instrumental Activity of Daily Living; CIRS: Cumulative Illness Rating Scale; GDS: 15-item Geriatric Depression Scale; TUG: Timed Up and Go test; HG: hand grip; FI: 40-item Frailty Index.

**Table 2:** Univariate and multivariate Cox proportional hazards models for death.

	Univariate		Multivariate Model 1		Multivariate Model 2	
	HR (95% CI)	*p*-Value	HR (95% CI)	*p*-Value	HR (95% CI)	*p*-Value
Age at diagnosis	1.035 (0.992–1.080)	0.116	1.013 (0.942–1.091)	0.723	0.993 (0.939–1.049)	0.792
Smoking	1.008 (1.002–1.015)	0.012	1.002 (0.993–1.012)	0.639		
T stage (ref T1-2)	1.965 (0.979–3.940)	0.057				
N stage (ref 0) 1	1.801 (0.238–13.608)	0.568				
N 2-3	1.303 (0.717–2.367)	0.385				
Tumor stage	1.557 (0.958–2.580)	0.074	2.278 (1.225–4.236)	0.009	1.728 (1.023–2.917)	0.041
Concomitant chemotherapy	0.419 (0.214–0.820)	0.011	0.883 (0.313–2.488)	0.813	0.420 (0.176–0.999)	0.050
MMSE	0.965 (0.882–1.055)	0.435				
CDT	0.963 (0.786–1.179)	0.712				
MNA	0.883 (0.829–0.940)	<0.001	0.958 (0.862–1.063)	0.418		
Lost IADL	1.482 (1.288–1.706)	<0.001	1.451 (1.087–1.936)	0.012		
Barthel Index	0.948 (0.926–0.971)	<0.001	0.919 (0.850–0.995)	0.036		
CIRS severity	2.570 (1.218–5.423)	0.013	6.087 (0.720–51.448)	0.097		
CIRS comorbidity	1.206 (1.041–1.397)	0.013	0.786 (0.529–1.167)	0.233		
GDS	1.116 (1.032–1.207)	0.006	1.091 (0.937–1.272)	0.261		
TUG	1.063 (1.006–1.124)	0.031	0.922 (0.834–1.019)	0.113		
HG	0.971 (0.939–1.004)	0.084	0.994 (0.953–1.038)	0.797		
N of drugs	1.158 (1.054–1.271)	0.002	0.953 (0.781–1.165)	0.643		
EuroQoL	0.113 (0.028–0.451)	0.002	0.760 (0.055–10.507)	0.838		
FI (+0.1)	1.640 (1.33–2.022)	<0.001			1.478 (1.182–1.848)	<0.001
			Concordance: 0.742		Concordance: 0.690	

Abbreviation list: HR: hazard ratio; 95% CI: confidence interval; ref: reference category; TNM staging: Tumor, Node, and Metastasis staging; MMSE: Mini-Mental State Examination; CDT: Clock Drawing Test; MNA: Mini Nutritional Assessment; IADL: Instrumental Activity of Daily Living; CIRS: Cumulative Illness Rating Scale; GDS: 15-item Geriatric Depression Scale; TUG: Timed Up and Go test; HG: hand grip; FI: 40-item Frailty Index.

## Data Availability

The raw data supporting the conclusions of this article will be made available by the authors upon request.

## Supplementary Materials

The following supporting information can be downloaded at https://www.mdpi.com/article/10.3390/cancers16233939/s1; S1: The preoperative CGA and a frailty assessment according to the 40-items Frailty Index; S2: Frailty assessment; S3: Univariate and multivariate analysis with radiation-induced toxicity as the dependent variable, adjusted for age, stage, and concomitant chemotherapy. References [29,30,42,43,44] are cited in Supplementary Materials.
