# Supplementary material for: Frailty and Overall Survival of Older Patients Undergoing Radiotherapy for Head and Neck Cancer: A Prospective Analysis
Source: Cancers (Basel). 2024 Nov 25;16(23):3939. doi: 10.3390/cancers16233939 (PMC11639954; doi:10.3390/cancers16233939)
Supplement: Supplementary file 1 [file cancers-16-03939-s001.zip › cancers-3301802-supplementary.pdf]

Supplementary materials

S1:

Table S1

CGA and frailty assessment	Clinical domain	ITEMS	RANGE	CUT OFF
**MMSE**	Cognitive status	30	0-30	<25
**CDT**	Cognitive status	1	1-6	>1
**GDS**	Psychological status	15	0-15	>5
**IADL**	Functional status	8	0-8	≤7
**Barthel Index**	Functional status	10	0-100	<50
**MNA**	Nutritional status	18	0-30	<24
**CIRS severity**	Comorbidity	14	0-5	
**CIRS comorbidity**	Comorbidity	14	0-14	>3
**Polypharmacy**		-		≥5
**EQ-5D**	Quality of Life	5	0-1	-
**TUG**	Physical Performance	1	-	≥ 15 "
**FI**	Frailty	40	0-1	Fit ≤ 0.08<br>0.08<Pre-frail <0.25<br>Frail ≥ 0.25

Table S1: The preoperative CGA and a frailty assessment according to 40-items Frailty Index. Abbreviations: MMSE: Mini Mental State Examination; CDT: Clock Drawing Test; GDS: Geriatric Depression Scale; IADL: Instrumental Activities of Daily Living; MNA: Mini Nutritional Assessment; CIRS: Cumulative Illness Rating Scale comorbidity index; EQ-5D: EuroQol- 5 Dimension; TUG: Time Up and Go test; FI: 40-items Frailty Index.

S2: Frailty assessment

Frailty is the most problematic expression of population aging. Older persons are characterized by high clinical complexity, multiple chronic diseases with consequent polypharmacy, disabling conditions, and social issues. According to Clegg et al., frailty appears when the reserve capacity has decreased to a critically low point [1]. From a clinical perspective, this reduction of the reserve capacity of various physiological systems constitutes a condition of greater risk of adverse outcomes, such as falls, less mobility, less independence, hospitalization, disability, and death.

One of the most highly cited frameworks that operationalize the definitions of frailty is the deficit accumulation approach, termed as the Frailty Index (FI), described by Rockwood and Mitnitski in the Canadian Study of Health and Aging [2]. Initially, this model defined frailty based on 92 baseline variables, including health behaviors, functional limitations, diseases, physical and cognitive impairments, psychosocial risk factors, occurring with the aging process and plausibly contributing to poorer health states. The FI was a simple calculation of the presence or absence of each variable as a proportion of the total items considered in the computation [3]. With more deficits, the likelihood of frailty increases. Subsequent models tried to reduce the number of variables and studies have suggested that at least 20 deficits should be considered. Even so, an index with 30–40 variables was demonstrated to be sufficiently accurate for predicting adverse outcomes. The deficits contribute cumulatively to an increased risk of adverse events, supporting the concept of a progressive reduced homoeostatic reserve and homoeostatic failure that is essential to the notion of frailty [4]. The cumulative deficit model expresses the theory of a gradation of frailty with progressive accumulation of deficits over the course of life. So, the FI aims to incorporate the multidimensional nature of frailty, basing its consistency on the results of a Comprehensive Geriatric Assessment (CGA).

In our study, we employed the 40-item version of FI validated in Italian by Abete et al.[5].

1. Help bathing (yes = 1/no = 0)
2. Help dressing (yes = 1/no = 0)

3. Help getting in/out of chair (yes = 1/no = 0)
4. Help walking around house (yes = 1/no = 0)
5. Help eating (yes = 1/no = 0)
6. Help grooming (yes = 1/no = 0)
7. Help using toilet (yes = 1/no = 0)
8. Help up/down stairs (yes = 1/no = 0)
9. Help lifting 10 lbs (yes = 1/no = 0)
10. Help shopping (yes = 1/no = 0)
11. Help with housework (yes = 1/no = 0)
12. Help with meal preparations (yes = 1/no = 0)
13. Help taking medication (yes = 1/no = 0)
14. Help with finances (yes = 1/no = 0)
15. Lost more than 10 lbs in last year (yes = 1/no = 0)
16. Self-rating of health (poor = 1/fair = 0.75/good = 0.5/very good = 0.25/excellent = 0)
17. Health has changed in last year (yes = 1/no = 0)
18. Stayed in bed at least half the day due to health (in last month) (yes = 1/no = 0,%)
19. Cut down on usual activity (in last month) (yes = 1/no = 0, %)
20. Walk outside (yes = 1/no = 0, %)
21. Feel everything is an effort (most of time = 1/sometime = 0.5/rarely = 0, %)
22. Feel depressed (most of time = 1/sometime = 0.5/rarely = 0, %)
23. Feel happy (most of time = 1/sometime = 0.5/rarely = 0, %)
24. Social support (> 13 = 1/6–13 = 0.5/1–5 = 0)
25. Have trouble getting going (most of time = 1/sometime = 0.5/rarely = 0, %)
26. High blood pressure (yes = 1/suspected = 0.5/no = 0, %)
27. Heart attack (yes = 1/suspected = 0.5/no = 0, %)
28. CHF (yes = 1/suspected = 0.5/no = 0, %)
29. Stroke (yes = 1/suspected = 0.5/no = 0, %)
30. Cancer (yes = 1/suspected = 0.5/no = 0, %)
31. Diabetes (yes = 1/suspected = 0.5/no = 0, %)
32. Arthritis (yes = 1/suspected = 0.5/no = 0, %)
33. Chronic lung disease (yes = 1/suspected = 0.5/no = 0, %)
34. MMSE (< 10 = 1/11–17 = 0.75/18-20 = 0.5/20–24 = 0.25/> 24 = 0)
35. BMI (< 18.5, ≥ 30 = 1/25– < 30 = 0.5/18.5–24.9 = 0)
36. Peak Expiratory Flow (yes = 1/no = 0, %)
37. Shoulder strength (yes = 1/no = 0, %)
38. Grip strength (yes = 1/no = 0, %)
39. Mini Nutritional Assessment (< 17 = 1/17–23.5 = 0.5/24 = 0)
40. Rapid pace (> 10 = 1/≤ 10 = 0)

[1] A. Clegg, J. Young, S. Iliffe, M. O. Rikkert, e K. Rockwood, «Frailty in elderly people», *Lancet Lond. Engl.*, vol. 381, fasc. 9868, pp. 752–762, mar. 2013, doi: 10.1016/S0140-6736(12)62167-9.

[2] K. Rockwood e A. Mitnitski, «Frailty in Relation to the Accumulation of Deficits», *J. Gerontol. A. Biol. Sci. Med. Sci.*, vol. 62, fasc. 7, pp. 722–727, lug. 2007, doi: 10.1093/gerona/62.7.722.

[3] K. Rockwood e A. Mitnitski, «Frailty, fitness, and the mathematics of deficit accumulation», *Rev. Clin. Gerontol.*, vol. 17, fasc. 1, pp. 1–12, feb. 2007, doi: 10.1017/S0959259807002353.

[4] A. Mitnitski *et al.*, «Relative Fitness and Frailty of Elderly Men and Women in Developed Countries and Their Relationship with Mortality», *J. Am. Geriatr. Soc.*, vol. 53, fasc. 12, pp. 2184–2189, dic. 2005, doi: 10.1111/j.1532-5415.2005.00506.x.

[5] P. Abete *et al.*, «The Italian version of the "frailty index" based on deficits in health: a validation study», *Aging Clin. Exp. Res.*, vol. 29, fasc. 5, pp. 913–926, ott. 2017, doi: 10.1007/s40520-017-0793-9.

S3:

Table S3

	Univariate		Multivariate	
	OR (95% CI)	*p-value*	*OR (95% CI)*	*p-value*
**Age at diagnosis**	0.970 (0.912 – 1.027)	0.290	1.047 (0.967 – 1.137)	0.784
**Smoking**	0.994 (0.983 – 1.004)	0.272		
**Tumour stage**	1.525 (0.897 – 2.897)	0.149	1.383 (0.782 – 2.658)	0.264
**Concomitant chemotherapy**	2.323 (1.075 – 5.090)	0.033	1.914 (0.670 – 5.631)	0.287
**MMSE**	1.007 (0.893 – 1.141)	0.915		
**CDT**	1.021 (0.789 – 1.317)	0.869		
**MNA**	0.927 (0.840 – 1.018)	0.118		
**IADL**	1.107 (0.875 – 1.405)	0.390		
**Barthel Index**	1.002 (0.959 – 1.051)	0.087	0.965 (0.899 – 1.035)	0.299
**CIRS severity**	0.319 (0.091 – 0.993)	0.059	0.290 (0.013 – 4.218)	0.398
**CIRS comorbidity**	0.828 (0.664 – 1.016)	0.080	0.952 (0.582 – 1.603)	0.848
**GDS**	0.989 (0.872 – 1.115)	0.859		
**TUG**	0.946 (0.840 – 1.048)	0.032	0.907 (0.777 – 1.037)	0.182
**HG**	1.001 (0.960 – 1.044)	0.955		
**N of drugs**	0.930 (0.820 – 1.050)	0.251		
**EuroQoL**	2.756 (0.303 – 31.848)	0.386		
**At least 1 geriatric intervention**	1.474 (0.693 – 3.188)	0317		
**FI**	2.729 (0.132 – 55.540)	0.509		

Table S3. Univariate and multivariate analysis with radiation-induced toxicity as the dependent variable, adjusted for age, stage and concomitant chemotherapy. Abbreviation list: OR: Odds ratio; 95% CI: confidence interval; ref: reference category; TNM staging: Tumour, Node and Metastasis staging; MMSE: Mini-Mental State Examination; CDT: Clock Drawing Test; MNA: Mini Nutritional Assessment; IADL: Instrumental Activity of Daily Living; CIRS: Cumulative Illness Rating Scale; GDS: 15-item Geriatric Depression Scale; TUG: Timed Up and Go test; HG: Hand Grip; FI: 40-items Frailty Index.
